# At the Right Time: Temporal Precision in Personalised Medicine

**DOI:** 10.1111/1467-9566.70180

**Published:** 2026-04-05

**Authors:** Dominik Hofmann, Elena Esposito

**Affiliations:** ^1^ Faculty of Sociology, Bielefeld University Bielefeld Germany; ^2^ Department of Political and Social Sciences University of Bologna Bologna Italy

**Keywords:** algorithms, personalisation, precision medicine, prediction, synchronisation, time

## Abstract

Personalised medicine was initially heralded as delivering ‘the right drug to the right patient at the right time’. Although molecular precision has dominated recent developments, the temporal dimension has remained underexplored. This paper examines how temporal precision is emerging as a defining feature of next‐generation precision medicine, driven by algorithmic tools and multiomics data integration. Drawing on qualitative interviews with leading experts in personalised immunotherapy and chronic inflammatory disease (CID) medicine, we identify five ways in which temporal precision is reshaping therapeutic practice: extended prediction, timing, synchronisation, coordination of treatments and feedback effects. We show how personalised cancer vaccines and precision inflammation approaches rely on algorithmic assemblages to anticipate therapy effects, coordinate interventions and dynamically adapt treatment schedules. These innovations highlight a shift from molecular precision to personalisation that incorporates the evolving temporality of both disease and therapy, signalling a new development in precision medicine: Diseases are increasingly treated as dynamic processes, therapies as sequences of timed interventions and patients as embedded in feedback loops between body rhythms, pathology and treatment regimens.

## 1 Introduction

Personalised medicine today is no longer the same endeavour it was when the term gained traction after the completion of the Human Genome Project in 2003. That ‘the right drug for the right patient at the right time is the mantra of personalized medicine’ (Abrahams 2008) may still hold, but the meaning of this statement has undergone several shifts over time. The expression personalised medicine has mostly been replaced by ‘precision medicine’ (Jaccard et al. 2017; Erikainen and Chan 2019) and tends to refer primarily to an unprecedented focus on targeted therapies and diagnostic tools, using algorithmic procedures to identify biomarkers that can provide insights to craft individualised (i.e., precise) treatment plans.

The reference to the ‘right time’ has not received much attention in this development. In this paper, we argue that the temporal side of precision medicine shows signs of now re‐emerging as a central element in a new generation of innovative precision medical therapies. Focusing on recent developments in personalised cancer vaccines and precision inflammation medicine, we show that timing, synchronisation, anticipatory planning and prediction of therapy effects are gaining increasing importance in current research and development. Applied to the individual patient, such temporal precision fosters personalisation by adding a time dimension. We trace the causes of this development to the emergence of new algorithmic techniques in diagnosis and drug development.

Since the completion of the Human Genome Project, technological innovation—particularly in sequencing, molecular profiling and computational analytics—has been a principal driver of precision medicine's development and clinical uptake. In many cases, practising precision medicine has become tantamount to sequencing genomes with high‐throughput next‐generation sequencing (NGS) methods and comparing individual genetic variations with records from variant databases—where both NGS tools and databases are the offshoot of digital algorithms. The more recent move towards including multiomics (considering not only genomics but also epigenomics, transcriptomics, proteomics, exposomics etc.) is inseparable from developments in artificial intelligence enabling the integration of highly heterogeneous data types (Meunier and Herzog 2023). In this context, algorithmisation refers to the process through which biomedical practices are supported by computational procedures so that diagnoses, predictions and treatment decisions emerge from the interplay between formalised, data‐driven algorithms and clinical judgement. We emphasise how such models are interlinked, depend on each other's operations and together constitute a specific overarching logic. We argue that algorithmically powered precision medicine is now introducing more temporal aspects in multiomics research.

The research fields that serve as our empirical cases, personalised cancer vaccines and precision inflammation medicine (introduced in more detail below), represent some of the most cutting‐edge subfields of contemporary precision medicine. In both fields, the topic of temporal dynamics emerges as central. We explored them following two guiding questions: How is the formula ‘at the right time’ concretely operationalised? Why is it being given renewed emphasis right now? Our investigation highlights the importance of big data and algorithmisation, mediated through the management of increasing complexity of diseases, treatments and analytical technologies. Thus, this paper contributes to the growing literature on the effects of algorithmic involvement in medicine (Beam et al. 2023; Hoeyer 2019; Hajiheydari et al. 2025), suggesting that one such effect is what we term ‘temporal precision’.

In the next section, we present a broad outline of the temporal aspects of precision medicine and their connection to recent developments. The subsequent sections describe our research materials and methods and introduce our two fields of research, personalised immunotherapies and precision medicine for chronic inflammatory disease. In the results section, we present the different forms of innovative temporalisation and timing observed in the field, which are then discussed with regard to their contribution to personalisation and the role algorithms play in it. In the conclusions, we link these findings to the current development of precision medicine from a broader perspective.

## 2 Time and Timing in Clinical Medicine

Temporality is, in many respects, a constitutive aspect of illness and its treatment. Any state of illness is characterised by a sense of urgency, where relieving the uneasiness takes precedence over everything else, and an inability to provide this relief immediately. This is not only a personal problem for the ill person but also a social problem for the wider society whose routines are disrupted by the illness. Accordingly, the social function of medical institutions is to bring this disruption under control and, not least, to give it a proper temporal structure (Luhmann 2005). This takes its most evident form in the institution of the hospital, characterised by extremely strict and precise timing of routine processes, the omnipresence of time measurements and deadlines, and the fact that, unlike almost every other organisation, it operates continuously without interruption (Fox 1989, 153f).

Molecular medicine, with which we are concerned here, is exploring new aspects of temporality in the form of temporal *personalisation*, enabled by recent algorithmic technologies.^1^ Recent studies show that precision medicine is not only about molecular specificity but also about the active configuration of time—creating new rhythms of monitoring, intervention and anticipation that structure what counts as actionable, preventable or treatable (Gjødsbøl et al. 2021).

Some of these possibilities concern the *temporality of the body*, with new developments in *chronomedicine*. They are focused on finding the best timing for interventions (i.e., usually the right time of day), concerning both efficacy and tolerability. Although the approach of timing certain therapies has been investigated in trials since the 1970s, the field is currently gaining a new significance, thanks in particular to molecular findings on the circadian clock (the cell clock synchronising the various levels of the organism to a 24‐h rhythm) and the measurability of RNA expression levels (Rasmussen et al. 2022; Kaşkal et al. 2025).

A second set of possibilities of temporal personalisation regards the *temporality of pathology*. This development is connected with the recent algorithmic processing of increasingly longitudinal data. Precision medical tools such as temporal multiomics, time‐resolved NGS and especially ‘pseudo‐time’ in single‐cell technologies are used to measure both continuous and discrete disease progression at an unprecedented depth of resolution (e.g., subclonal tumour evolution, metastasis, autoimmunity and acquired resistance). Complex diseases are increasingly perceived as evolving processes with their own history that begins before onset and keeps evolving also in the chronic stage.

In this paper, we explore a third possibility of temporal personalisation: the *temporality of treatment*. It is, of course, tightly interconnected in a network of feedback effects with the other dimensions of temporality, but sociologically this is the most relevant aspect. It is not only concerned with bodies and the research on them but also concerned with the real‐life work of practitioners and the practical implementations of technologies in the health system.

## 3 Materials and Methods

The investigation presented here draws on scholarship in the sociology of medicine and of quantification, on close reading of scientific publications, on our attendance at several medical conferences on recent developments in precision medicine and on a thorough literature review, including academic publications and promotional materials (working papers, patents, newsletters, press releases, posters, flyers and website content) from various institutions in the field.

On the basis of this preliminary work, we identified two settings, *personalised therapeutic vaccination* (PTV) and *chronic inflammatory diseases* (CID), where the debate about the temporal aspects of precision medicine is particularly lively, frequent and innovative—as confirmed by conference presentations and by our informal conversations with practitioners in the field. Both settings connect advances in targeted therapy research with developments in immunotherapy and use multiomics sequencing and other innovations relying on algorithmic tools for molecular data analysis. In both cases, *personalisation* implies making use of molecular features of the individual body, building in the case of personalised vaccines on characteristics of the concrete tumour and in inflammation medicine on antigens produced naturally. This approach goes beyond personalisation as the mere assignment of patients to treatment groups based on statistical molecular markers, which characterised many earlier forms of precision medicine.

After a detailed assessment of their respective methods and technologies, we concluded that the two settings could be productively investigated together, focusing on their similarities and differences. We recruited interview partners through snowball sampling, starting from the network of contacts established at conferences we attended. Despite the difficulty in establishing in‐depth contacts with people working in these sectors, we have been able to conduct eight semi‐structured qualitative interviews with high‐ranking medical researchers, practitioners and bioinformaticians working either in personalised immunotherapy research or in precision medicine for chronic inflammatory disease. Because of the central role of our interlocutors, the meetings proved to be highly informative.

More precisely, three of our interview partners work on inflammation (I1–3), five on personalised vaccines and/or cell transfer therapy (I4–8). Five of our interviewees work in academia or university hospitals, whereas the other three operate in companies that develop therapeutic approaches.^2^ The majority of interviews were conducted in German, and direct quotes from them in the following sections are our translations. We used the AI‐powered noScribe software to create transcription files, which we then manually revised, corrected and anonymised. All the software works locally; at no point was any data sent to the internet.

The interviews lasted between 27 and 59 min (mean: 41 min) and were mainly focused on the use and exact functioning of algorithms and on different aspects of personalisation. The issue of ‘temporal precision’ or timing often emerged unprompted, although we did encourage our interviewees to elaborate on it. In the analysis of our materials, we paid special attention to two aspects: the importance of algorithms (tasks fulfilled by them, data types used and importance for the workflow) and timing, and the development over time of the fields of personalised immune therapies and precision inflammation medicine. By asking our interview partners about the order in which key innovations emerged, we gained clearer insight into the extent to which current developments are rooted in technological developments—particularly in algorithmic technologies. Our interviewees included people regarded as pioneers in their respective field, which made them particularly well suited as informants on the topics of our research.

## 4 Two Settings: Personalised Immune Therapies and Precision Inflammation Medicine

As anticipated above, our research is focused on two highly innovative settings within precision medicine. Our first setting is *personalised therapeutic vaccination* (PTV), a specific form of immunotherapy currently introduced as part of a new generation of oncological treatments besides the traditional triad of surgery, radiotherapy and chemotherapy. Although vaccinations are commonly seen as preventive measures, in this case, they function as therapeutic modalities tailored to an existing tumour, thus personalising treatment (Montin et al. 2024).

Encompassing a variety of approaches, immunotherapy is based on the idea of ‘programming’ the patient's immune system to make it fight a pathology that has, up to that point, successfully evaded the immune response. Two such therapeutic modalities have already been in use for some years: immune checkpoint inhibitors and CAR‐T‐cell therapy. Although highly innovative, they are not individualised in the strict sense.^3^ We focus on therapeutic cancer vaccines (Blass and Ott 2021; Chi et al. 2024) because they are instead produced in a more strongly personalised way by identifying the characteristics of the patient's specific tumour cells (peptides present on the surface) and programming immune cells to target these epitopes. Antibodies are introduced into the body through a delivery mechanism (mRNA, DNA, peptides or bacteria) to activate the immune cells, enhanced by an adjuvant that boosts the immune response and potentially accompanied by checkpoint inhibitors that enable T cells to engage more effectively. In contrast to T‐cell therapy, these antibodies are not genetically modified but selected from among particularly promising neoantigens given the individual tumour signature. Algorithmic pipelines are employed for the crucial tasks of predicting which peptides are particularly effective targets (‘neoantigen discovery’) and selecting candidate antibodies (‘neoantigen prioritisation’) (Haen et al. 2020). Universities and companies developing the vaccines employ large bioinformatics units to run and manage them.

The second setting we analysed goes beyond the two paradigmatic disease types in the field: cancer and rare mendelian disease. *Chronic inflammatory diseases* (CID) include, for example, inflammatory bowel disease, lupus, vasculitis, Type 1 diabetes and rheumatoid arthritis. These illnesses, though traditionally classified into subcategories such as autoimmune diseases and chronic destructive diseases, are now unified in new data‐intensive approaches that transcend traditional taxonomies.^4^ The healthcare system records a very high number of cases involving CIDs, with equally high associated costs. Algorithmic prediction is used in this field for several tasks: biomarker discovery, multiomic disease profiling and forecast of disease progression. In many respects, these developments follow the footsteps of precision oncology, where the genomic component (DNA mutations), however, has significantly greater penetrance (Schultze et al. 2018). As a result of the relatively lower importance of genomic data, in CID precision medicine, more emphasis is placed on the integration of highly heterogeneous data.

Our two settings are highly representative of current trends in precision medicine: It is becoming ‘postgenomic’ (Müller‐Wille and Rheinberger 2009; Richardson and Stevens 2015), integrates several levels of multimodal, multilevel data and is extending many institutional achievements of precision oncology to other fields. They are both developed in specialised health centres in large hospitals and in pharmacogenomic companies. They tend to be organised in networks (consortia, collaborations) to distribute tasks between specialised teams and to secure access to extensive databases. One of our examples illustrates a development moving ‘inwards’ from the established field of precision oncology towards greater precision and personalisation, whereas the other reflects a development moving ‘outwards’, expanding the lessons learnt from oncology to other fields.

Given that cancer and chronic inflammatory diseases are among the most prevalent and costly conditions in Western health systems and that mRNA vaccination and T‐cell regulation are major current trends in medicine—underscored by the consecutive Nobel Prizes in physiology or medicine awarded over the past 3 years—our research focus is representative of the cutting edge of precision medicine.

## 5 Results

The analysis of our material has revealed five recurring topics, underscoring the heightened importance of temporal precision both in chronic inflammation medicine and in individualised immunotherapy. The descriptive presentation in this section prepares the discussion that we will develop in the following one.

### 5.1 The Meaning of Prediction: From Risk Assessment to Changes in Disease State

Both areas of precision medicine we observed are part of a move from generic risk prediction towards prediction focused on confirmed patients. According to a precision gastroenterologist we interviewed (I2), CID medicine becomes ‘precision’ medicine when it is about determining ‘how to assign therapies in the case of existing disease and also trying to understand in the case of existing disease why someone may have a severe or a mild disease course’. A central aim, for him, is to answer the question ‘Is it possible to predict the heterogeneity of the disease?’.

Thus, in the opinion of a clinical microbiologist,

if you talk to a patient today, it must be said that if you start this therapy, the probability that it will still be working well after a year, in the sense that there are no symptoms, is around 25 to 30 percent. […] How can you actually navigate that? Because that's independent of early [detection] and risk […]. That's a different prediction. Namely, it's the prediction of finding the right therapy for a disease that has already occurred or for a diagnosis that has already occurred. And it's basically a bit like cancer, except that with cancer you look for the mutations and can deduce a little bit and see if the surface proteins against the targeted therapy are even there. And then the pathologist says yes and then the therapy works better and so on. And we don't have that at all with our disease (I1).^5^

An additional step of temporalisation is to predict not only changes in disease course but also in therapy response. In both cancer and CID medicine, initial therapy effects often do not last long and treatment failure occurs over time. As the disease adapts, the treatment must also do the same. In our experimental settings, as therapy becomes a process stretched out in time, an initial prediction about drug effectiveness may be complemented by updated follow‐up predictions:

These are the predictions that we make or that we are working on: after two months or after two weeks, we look to see if there are biomarkers that can predict that it will still be effective after a year. And if these biomarkers are negative, then we don't go down the usual route of talking to the patient again, ah, it was a bit better and can we try it for another six weeks, but instead we really aggressively change the therapy, because we know that the longer a patient is poorly treated, […] ‘poorly’ in the sense of ‘insufficiently’, the more difficult it is to switch to the next therapy. And that is the prediction algorithm we have, so to speak (I1).

To cope with this broad variety of prediction tasks, the professionals we interviewed rely on combinations of algorithmic tools, collaborating closely with bioinformatics units. These algorithmic assemblages are channelled into ‘pipelines’. This is most evident in the case of vaccine development, where successive steps in the process—genomic sequencing, neoantigen prediction, neoantigen selection and drug design—build on each other, using data from different molecular levels.^6^ Algorithmic packages are applied in each of these steps, sometimes to validate in silico nonalgorithmically obtained experimental results or vice versa (Haen et al. 2020, 607). Modular algorithmic pipelines are central also in precision CID medicine but are geared towards treatment recommendations rather than drug development.

These developments require new types of data and data acquisition. To enable predictions of changes in disease course and treatment response, the data collected in precision medicine are becoming less static and more longitudinal. According to a bioinformatician we interviewed, ‘all this time course data is usually always collected for treatment planning purposes’ (I3), and ‘time‐course data could be collected as part of the normal clinical routine, if, for example, daily blood drawing was performed at different times of the day’ (Hesse et al. 2020, 22). This leads to our second aspect of temporalisation.

### 5.2 Timed Interventions

An increasingly comprehensive recording of health data makes more precisely timed interventions possible. Although costs and intrusiveness set limits to the exhaustiveness of data acquisition, technologies are steadily becoming cheaper and less intrusive. Our interview partners cited long lists of the different data types they use. Especially in CID medicine, ‘clinical data’ (patient‐reported outcomes, endoscopy, histological analysis and pharmacokinetic assessment of concentration rates in the blood) are supplemented by several molecular data levels: from the transcriptome, methylome, microbiome and the metabolome.

A first element of such data acquisition concerns *screening before the therapy*, on the individual as well as on the populational level. As one programme director in precision CID reports (I1), part of the work consists in ‘looking at large cohorts of relatives even before diagnosis’ to assess ‘how certain molecules are different’. To get a picture, ‘they are traced for years’. Because only a few of the people in these cohorts will develop a given disease, one can ask ‘how do these few look different shortly before diagnosis than those who live at a certain risk but do not get the disease. And from this in turn, of course, we hope to derive prediction mechanisms, but especially intervention options as well’.

In both areas we investigated, screening implies an assessment of the common and the individual patient's ‘immune repertoire’, that is, the landscape of T and B cells available in the body. Although predictive and therapeutic mechanisms are developed based on these large‐scale data, one immunologist explained that ‘we also use this technology beyond that, for example to say how immunocompetent someone is. To get an individualised impression of how well someone will react to a vaccine when we then administer it’ (I7).

A second element is the *monitoring* of the immune response *once the treatment is underway*. In CID medicine, this implies data collection to predict critical events:

There are small devices where you take a few drops of blood, in which we can analyse our biomarkers. And this is the kind of home‐based sampling that we do with people in these cohorts for rheumatoid arthritis and chronic inflammatory bowel disease, where a few drops of blood are analysed very densely over time for such markers that systematically detect, such warning signals, so to speak before the emergence of the next flare, before the emergence of the next wave of inflammation, so that you can tell the physician […] that's our idea, there's a simple test that predicts an impending flair‐up two weeks before the inflammation, so that you can either call the patient in and discuss whether there are ways of tightening up the diagnosis even before symptoms are actually present (I1).

Some of the prediction tools developed for these purposes integrate the temporal dimension directly into the *clinical endpoint*, as explained by one bioinformatician developing such a prediction model: ‘it's a combined endpoint, you have time and you have a probability and you have a time horizon for which you get a prediction […] you can say, okay yes, for the next 365 days, 20 percent relapse’ (I3). Additionally, ‘for patients with autoimmune diseases or those undergoing organ transplantation, the timing of immunosuppressive drugs can be crucial. Administering these drugs at times when the immune system is less active (e.g., late evening or night) can enhance their efficacy and reduce the risk of rejection or flare‐ups’ (Kaşkal et al. 2025, 10).

All our interview partners involved in the development of a new type of vaccines told us that although they are not focused on continuous patient care, monitoring the immune response is standard for them, as part of the trials testing the effectiveness of the treatment. Particularly interesting is that these activities occasionally extend beyond such assessment into the clinic, again with a temporal index:

we have taken a few […] measurements where we have said that we look at an individual over a long period of time and the outcome of our biomarker was constant over time. This means that *one* measurement is actually sufficient to be able to say that an individual is reacting well or badly. Where monitoring must then take place is when the titer breaks down. We still have no idea for *which* profile we would expect a poor response to occur or that a response will not last as long (I7).

These are clinical settings, but simultaneously also sites of research and development. Reflecting on future trajectories, the same interviewee suggests the temporal customisation mentioned above might complement or even replace other forms of personalisation: ‘Actually with vaccines, at least when designing them, you would tend to look for an approach that does not have to be personalized. That is much more time‐consuming. [You must ensure] that the vaccine be declared safe, that it can go into clinical trials, get it through the trials. Every modification has to be toxicologically tested. That’s why it […] makes the most sense to standardize the vaccine, but then to individualize the frequency of application, for example’ (I7).

Although generally positive about the theoretical possibility of such a system, our respondents tended to doubt its practical viability—at least given the current state of technological development for monitoring and prediction. As one representative of a pharmacological company objected: ‘You would have to take an immune sample every time and see how high the peak is now […]. You could theoretically do that, yes. But whether the patients would go along with it is another question. You need a relatively large amount of blood for this immune test’ (I6). Another caveat, added by a bioinformatician (I8), is that currently the limited amount of data would not allow to take these types of therapeutic decisions, especially because heavily regulated machine learning might be involved.

A major part of the arguments about the viability of such approaches for exactly timed interventions is based on *technological* considerations. Screening, monitoring and precision in the timing of interventions do not rely on single innovations but on a wide range of technological developments combined in creative manners: From the easy‐to‐use wearable technologies, we encountered in different CID medicine projects to the cutting‐edge single‐cell sequencing technologies tracing tumour evolution with ultrahigh resolution to the techniques for creating reliable synthetic data to complement missing values in noisy longitudinal data sets (I3). Temporalisation is anticipated on numerous levels which are connected by assemblages of algorithms.

### 5.3 Stages of Disease: Prevention/Intervention and Curation/Chronicisation

The two disease types we investigate differ significantly in terms of urgency, because cancer leaves little time for treatment planning. Nevertheless, one communality is the aim of turning the disease into a controlled entity with the help of the immune system, thus gaining time. CID medicine is explicitly inspired by oncology in this regard: ‘For those who have the disease, it will be about controlling the disease to the best of our ability. There is the nice oncological term, minimal residual disease, and we are currently trying to propagate this also for inflammation’ (I1).

Another aspect common to both areas is a growing emphasis on the very early phase after the diagnosis. According to a gastroenterologist we interviewed (I2), ‘*early* management of the diseases is sensible and there is currently a strong movement towards pursuing a top‐down therapy principle, meaning intervening very intensely at an early stage with the chance of long‐term control’. Similarly, for cancer vaccines, ‘what makes sense is that you vaccinate relatively intensively at the beginning so that you have a relatively high response’ (I6). Interestingly, this focus on the early phase concerns not only therapy but also research. In the words of a molecular biologist working on CID (I1), ‘it is precisely in this early phase that we are interested in, starting or trying to understand […] the very early phase, that is, around the diagnosis, what actually characterizes it and can we possibly change this heterogeneity in the sense of tertiary prevention in the direction of severe progression’.

This tertiary prevention is distinguished from prevention before the onset of symptoms, also for legal reasons. As the same interviewee (I1) told us:

what is of course much more radical is to actually look at individuals who may or may not be at risk and look for such markers, a manifestation of the disease, even though the diagnostic criteria specified […] according to guidelines are not present. This is a completely different matter, because you are not allowed to treat them just like that. Because the treatment of a theoretically existing risk cannot *really* be justified either in terms of health economics or ethics.

This raises thorny issues of risk prediction. Because risk factors, markers and predictors have entered the arena of health and medicine, both ‘over‐treatment’ and ‘clinical inertia’ (Phillips et al. 2001) are problematised, and ‘temporal uncertainty’ is on the rise (B. Hofmann 2023).^7^ Practitioners are in a dilemma because, on the one hand, the possible interventions for patients ‘at risk’ are limited, whereas, on the other hand, the risk status warrants some form of action. In our settings, the reaction tends to be to prioritise tertiary prevention over prediction for presymptomatic patients.

What our respondents in CID do in these cases is a ‘low‐threshold intervention’ (a nutritional adaptation in this case) aimed at controlling the temporality of the disease ‘in patients who have an early diagnosis and, let’s say, a mild course or for whom things are calm for the time being, in order to see whether it is possible to change the length of time until the next episode with this kind of targeted nutritional therapy, so that it can then, because it is a vitamin, possibly also be rolled out in this risk area’.

Algorithmic technologies are crucial not only for these types of prediction but also because they exponentially shorten turnaround times for data analysis. Cancer vaccines and cell transfer therapies are only conceivable because algorithms reading molecular sequences and selecting candidate antibodies help deliver the personally tailored drug very quickly (about 4 weeks from initial screening to completion of first treatment in our cases)—a major achievement owing to computing power.

### 5.4 Temporal Coordination of Treatments

Both our areas of investigation go far beyond analysing variants in DNA sequencing data and marking driver mutations, like most applied precision medicine up to now. Being focused on the immune system, they combine several treatment approaches into a therapeutic regime.^8^ This also requires *temporal coordination of treatments*. As Nelde et al. (2021, 5) explain for therapeutic vaccines, ‘for selection of the optimal vaccination time point, potential concomitant therapies must be taken into account as these drugs can influence the outcome and efficacy of peptide vaccination […]. Furthermore, the vaccination schedule, including primary and boost vaccinations, may have an impact on the effectiveness and duration of the antitumour T cell response’. Accordingly, a researcher working on the development of such vaccines told us that ‘some of the new targeted therapies are really good, but they may do, in the future, the early heavy lifting in terms of getting rid of the bulky mass of the tumour, and then you can imagine a scenario where the immunotherapies then are the maintenance. […] because you’re not going to get rid of every tumour cell, there’s going to be some circulating, and [the goal is] being able to prevent a reoccurrence’ (I5).

This innovative coordination of treatments is connected with other forms of temporality that we discussed in the previous sections. It involves, on the one hand, the coordination across different disease stages:

so far it looks like people who already have certain cancer‐inducing mutations, i.e. BRCA1 or something like that, could perhaps be vaccinated beforehand. But if the cancer has already occurred in a person, then I would definitely do it as early as possible and preferably after the surgical resection of the tumour, which is often necessary, and then you can immediately see what the right antigens are based on the genetic material. And I would start this vaccination as soon as possible after the resection, after the operation, as a prophylaxis against recurrence, i.e. to prevent the tumour from coming back (I4).

On the other hand, the coordination also concerns the timing of booster vaccinations:^9^

And then we saw in the first study that after a certain period of time, which was about three weeks, the immune response slowly decreases again. In other words, the assumption that you vaccinate a patient now and they are then protected for life doesn't work that way. And then we considered, well, what could be a sensible period of time. And these three or four weeks are then also, I say, always associated with advantages and disadvantages, (…) they are then also relatively, let's say, consistent, for example with the administration schedule of some checkpoint inhibitors (I6).

### 5.5 Data Feedback Loops

Because many therapies that initially showed positive effects lose their effectiveness over time, several of our respondents discussed the possibility of even more dynamic circles of mutual adaptation between therapy and disease. Such a setup would require a complex data infrastructure that is not compatible with current data protection standards. Nevertheless, one can already observe some basic facilities hinting at such a development. As we saw above, sometimes acquired resistance is already predicted, and some of the prediction models are already dynamic: ‘so you have the history and then maybe you enter the latest biomarkers again, the latest values from the study and then […] you get an updated probability for the new time horizon’ (I3). As patient data are updated as the person moves through the trajectory of treatment, the changes since a last prediction feed back into the next one.

## 6 Discussion: Temporal Personalisation and the Role of Algorithms

Across both fields we studied—personalised immunotherapies and precision inflammation medicine—temporal reasoning is no longer a secondary consideration but a central axis along which personalisation unfolds. Our findings show that precision medicine is moving beyond its initial emphasis on molecular specificity towards a multidimensional form of personalisation in which time becomes a key variable for making diseases manageable and treatments adaptive. Our results suggest what this might mean in practice.

### 6.1 Integrating Molecular Precision With Temporal Precision

Precision medicine is commonly associated with tailoring interventions to the molecular characteristics of an individual patient. Our interviews reveal that now also considerations about the timing of clinical actions are becoming increasingly personalised: when—and how often—a therapy should be administered. Prediction models anticipate not only the likelihood of disease but also the expected trajectory of a condition already underway and the durability of a therapy that has already begun (Armstrong 2019; Cambrosio et al. 2021). These models are embedded into routine workflows, producing a dynamic management of predictions and updates: when to intensify treatment, when to switch drugs proactively and when a booster or second‐line intervention becomes necessary. This is how ‘the right time’ might be operationalised: the imperative to act as early as possible—long prevalent especially in oncology, where every second of inaction can allow the disease to spread—is not being abandoned. On the contrary, it is being further expanded aggressively and adopted in the CID setting. However, the points in time used to trigger new interventions are becoming more numerous and precise.

### 6.2 Temporal Coordination in Complex Therapeutic Regimes

A second implication of our findings concerns the increasing need to coordinate multiple interventions over time. Both personalised vaccines and CID therapies are rarely used in isolation; they are part of complex regimens that combine different interventions. Our findings show that these modalities must be synchronised in a personalised way. We have seen this, for example, in the temporal coordination of treatment regimens or in the introduction of time variables as clinical endpoints. In other cases, the timing of administration of a ‘warehouse drug’ is adapted to the single case, so that temporal personalisation substitutes for biomolecular personalisation of the drug agent. The emphasis on the ‘complexity’ of treatment regimens in the new generation of precision medicine, aimed at multifaceted illnesses such as cancer and chronic inflammatory diseases, reflects a notion of complexity centred on the integration of multimodal, high‐dimensional data. This emphasis coincides with a development in bioinformatics that makes such data integration technically feasible, promoted under the label of ‘systems medicine’ (Stevens 2015). When understanding disease and designing treatments increasingly rely on meticulously calibrated combinations of modalities, heightened temporal precision becomes both necessary and possible.

### 6.3 Reconfiguring Prevention and Intervention

Our data challenge the widespread sociological claim that the boundary between prevention and intervention is getting blurred (Aronowitz 2009, 2015; Giroux 2022; Baumgartner 2021). The claim relies on the observation that, in the settings we observed, many interventions aim at hindering early‐onset disease from getting worse, thus falling within the field traditionally defined as tertiary prevention (Gordon 1983). With such a broad definition, however, almost any medical action could be framed as preventive. In a more restricted sense, the boundary between intervention and prevention concerns not only the temporal order but also the fact that specific interventions only take place in hospitals or other medical institutions, whereas prevention addresses a broader societal context and tends to be more general. In the settings we studied, these boundaries remain largely intact. Tertiary prevention measures (such as dietary supplementation) for patients at risk of deterioration are not particularly specific. And prevention still happens in the environment: individuals that were not already patients were not enrolled. For the time being, rather than changing the logic of the distinction between prevention and intervention, timing is introduced as an *alternative* to it. We observe a *dynamic interweaving* of preventive and interventive logics that does not blur the border between them but rather makes it sharper^10^: intervening early in a diagnosed disease to delay worsening or the next flare or designing treatments that operate simultaneously as therapy and prophylaxis against recurrence. This interweaving is possible only when diseases are conceptualised as evolving processes rather than static states.

### 6.4 Performative Production of Time

In the practices we observed, temporal precision is not merely a reflection of biological temporality; it is produced through algorithmic assemblages. Each step in the pipeline we describe—data acquisition, monitoring of the reaction, determination of endpoints and prediction of therapy response—generates a temporal map onto which clinical decisions can be projected. In this sense, algorithms do not simply detect temporal patterns; they actively constitute the temporal structure through which disease and therapy become actionable. This performativity has practical implications. Monitoring regimes create new expectations for what counts as an early signal. And modular pipelines generate temporal dependencies between analytical tools, contributing to path‐dependent infrastructures that privilege certain rhythms of monitoring and intervention.

## 7 Conclusions

Our research in two innovative fields of cutting‐edge precision medicine shows that the temporal aspect of precision medicine, seemingly neglected in its early development, is in fact becoming a crucial component of the transformation of medical practice. Temporal precision emerges as a central axis of precision medicine, with the potential to reshape how diagnoses are made, therapies are timed and patient bodies are integrated into therapeutic processes.

This development is inherently connected with the impact of digital technologies. The use of longitudinal data in the way described above, for example, is a necessary condition for temporal precision and was simply inconceivable a decade ago. Attributing this change solely to technological innovation, however, would be simplistic. The various elements contributing to increasing temporal precision are diverse and lack a single origin or explanation. As is often the case in medicine, technological, epistemological and infrastructural developments continually feed into one another. To grasp their interconnections, it is useful to consider the field of precision medicine as a whole.

In its first stage, precision medicine was already highly dependent on algorithmic technology but not yet concerned with temporal aspects of disease and treatment. The reason can be largely traced back to the pivotal position of oncology and rare‐disease medicine in its development (Fleck 2022; Plutynski 2022). Patients who gained access to innovative treatments were mostly individuals for whom everything else had failed or no alternative was available (Bogicevic and Svendsen 2021). Given the urgency of the cases, the focus rested on diagnostic identification of driver mutations, at which targeted therapies could then be aimed.^11^

The two cases presented in this paper can be seen as representative of a new phase in the development of precision medicine, in which the area of application is expanding to new pathologies with broader time horizons and temporal precision is being added alongside molecular precision. The new relevance of the reference to the ‘right time’ in the formula originally used to describe the algorithmic personalisation of medicine thus might signal a fundamental change. What we describe as temporal precision appears as a key organising principle in the development of a precision medicine that is moving beyond the primacy of genomic analysis towards multiomics and algorithmic data integration. This would mark a departure from the dominant temporal logics of both traditional clinical medicine and the first wave of precision medicine. Although conventional care has long operated through episodic encounters and reactive interventions, and early precision medicine focused primarily on static molecular profiles, the emerging forms described in this paper treat disease as a dynamically evolving process. This could imply that precision medicine does not simply tailor treatments to individual cases but also manages the unfolding of illness across multiple timescales.

Precision medicine, moreover, is getting more integrated with other existing forms of clinical practice, as signalled by our exemplary settings, both relying heavily on the inclusion of the patient's immune system in the fight against disease. Although precision medicine is still an avant‐garde endeavour far from comprehensive health service provision, calls for more vigorous ‘translation’ from bench to bedside are gaining urgency (Evans et al. 2024), not least because of the biomedical industry's demand for returns on its investments. To achieve this translation, innovations from precision medicine are being integrated with long‐standing structures, routines and practices in the broader field of health provision, from hospital schedules to clinical trial planning. Both personalised immunotherapies and precision CID medicine are steps in this direction. As a result, temporal precision may not only reconfigure how diseases are known and acted upon but also transform practices of care, redistributing tasks and forms of expertise across the clinical system.

## Author Contributions

**Dominik Hofmann:** conceptualisation, investigation, writing – original draft, formal analysis, data curation. **Elena Esposito:** funding acquisition, writing – review and editing, project administration, supervision.

## Funding

This work was supported by the European Research Council (ERC) under Advanced Research Project PREDICT no. 833749.

## Conflicts of Interest

The authors declare no conflicts of interest.

## Data Availability

The data that support the findings of this study are available on request from the corresponding author. The data are not publicly available due to privacy or ethical restrictions.

## Endnotes

^1^
The emergence of molecular medicine has been observed as a change in the temporal exceptionality of illness. Geneticisation was expected to turn everybody into a patient: The individuals not currently under treatment were to be considered ‘patients‐in‐the‐waiting’ (Rosenberg 2007).

^2^
Because most our informants' companies and research divisions are developing several therapeutic products and/or medical decision‐making strategies simultaneously, this helped us identify the most relevant aspects for our interview questions. We often asked interviewees to explain or clarify claims from these promotional materials.

^3^
In CAR‐T‐cell therapy, antibodies from the patient are extracted, genetically modified and reinserted into the body. The template antibodies stem from the individual patient.

^4^
Just as in precision oncology, where taxonomic histology loses importance for tumour‐agnostic variant interpretation.

^5^
‘Targeted therapies’, which have been the most important additions to oncology in terms of molecular biological drug development since the 2000s, have recently also been introduced in CID medicine. Contrary to oncology, however, there are not yet clear biomarkers guiding the selection of these drugs for treatment.

^6^
To give an idea of the scale of algorithmic involvement: Sequencing tasks encompass whole‐exome and RNA sequencing and data integration and can be subdivided into ‘reads filtering’, ‘reads mapping’, ‘variant calling’ and ‘variant annotation’, all of which apply various algorithmic tools (e.g., FasTC, NGSQC, FASTX, Picard, RSeQC, Qualimap, edgeR, baySeq, EBSeq, DESeq and maSigPro). Regarding antigen selection, in the exemplary case of cancer vaccines using peptides as vectors, models were originally trained with peptide‐binding data from databases such as the Immune Epitope Database (IEDB) and are recently being optimised with further data types, including the HLA ligandome (MAPTAC, MixMHC2pred and NetMHCIIpan). Standardised algorithmic pipelines for this purpose are called, for example, CloudNeo, pVAC‐Seq, MuPeXI and NeoPredPipe.

^7^
As the sociology of risk and uncertainty (Beck 1992; Luhmann 2002) shows, risks tend to multiply because dangers are turned into risks whenever decisions are involved and inaction is also a decision.

^8^
A person working on the development of commercial therapeutic vaccines pointed out to us the resulting difficulty of proving that positive treatment effects are actually the result of the vaccine and not the effects of accompanying immunomodulators.

^9^
As is the case for all our citations taken from the setting of personalised vaccines, these treatments are still in the development phase. The observations stem from ongoing clinical trials in which they are currently tested.

^10^
As it also happens in several similar cases: cf. D. Hofmann and Esposito 2025.

^11^
We are forced to use broad strokes for painting the picture presented in this section. We describe a very general development to which there are many empirical exceptions. Also, in saying that the focus shifted, we do not mean to imply that more traditional ‘genetic medicine’ has disappeared.
